# Microstructural changes are coincident with the improvement of clinical symptoms in surgically treated compressed nerve roots

**DOI:** 10.1038/srep44678

**Published:** 2017-03-15

**Authors:** Weifei Wu, Jie Liang, Ying Chen, Aihua Chen, Yongde Wu, Zong Yang

**Affiliations:** 1 Department of Orthopedics, the People’s Hospital of Three Gorges University the First People’s Hospital of Yichang, Hubei, China; 2 Department of Nephrology, the People’s Hospital of Three Gorges University the First People’s Hospital of Yichang, Hubei, China; 3 Department of Radiology, the People’s Hospital of Three Gorges University the First People’s Hospital of Yichang, Hubei, China

## Abstract

Diffusion tensor imaging (DTI) has been widely used to visualize peripheral nerves, but the microstructure of compressed nerve roots can be assessed using DTI. However, there are no data regarding the association among microstructural changes evaluated using DTI, the symptoms assessed using the Oswestry Disability Index (ODI) and the duration of symptoms after surgery in patients with lumbar disc herniation (LDH). Thirty patients with unilateral radiculopathy were investigated using DTI. The changes in the mean fractional anisotropy (FA) and the apparent diffusion coefficient (ADC) values as well as the correlation between these changes and the severity and duration of the clinical symptoms were investigated before and at least one month after surgery. The FA values were significantly increased after surgical treatment (p < 0.0001). Both the ADC and ODI values were noticeably decreased (p < 0.0001). A strong positive correlation between the preoperative and postoperative DTI parameters (p < 0.0001) as well as between the preoperative ODI and postoperative ODI/ODI changes (p < 0.0001) were found. In addition, there was a significant positive correlation between the changes in the DTI parameters and changes in the ODI (p < 0.0001). This preliminary study suggests it may be possible to use DTI to diagnose, quantitatively evaluate and follow-up patients with LDH.

Lumbar disc herniation (LDH) entrapping a nerve root, which leads to lower back and leg pain, is common. Magnetic resonance imaging (MRI), which is often used to diagnose disc herniation, offers considerable information about the location and size of the disc herniation and as well as its mass effect on the nerve roots^1^,^2^,^3^. However, inconsistencies between the symptoms and the degree of nerve root compression seen on conventional MRI are continually observed. In addition, some studies reported symptom-free people with nerve root compression^4^. Therefore, it is difficult to use MRI to understand the cause of the pain and numbness. Moreover, MRI cannot provide a quantitative assessment of nerve root damage.

Diffuen widely used in the clinicsion-weighted imaging (DWI) can provide valuable information about the microstructure of the tissues by capturing the diffusion of water molecules in the restricted intracellular or extracellular fluids in the tissues. DWI has be to diagnose central nervous system diseases, such as acute brain stroke^5^. DWI is also used to evaluate peripheral nerve compression disorders, including nerve root compression by LDH^6^,^7^. The apparent diffusion coefficient (ADC) values from DWI can be used to determine the quantitative diffusion, and the ADC is useful as a differential diagnostic index for a variety of disorders.

However, DWI is influenced not only by the ease of diffusion of water molecules but also by the direction of the diffusion. This is because the axonal cell membrane and the myelin sheath surrounding the nerve fibres prevent diffusion in the direction perpendicular to their fascicles, resulting in isotropy of the diffusion of water molecules being lost^8^,^9^. This phenomenon is known as diffusion anisotropy, and selectively recording this information is referred to as diffusion tensor imaging (DTI). DTI is the only method that can give an indirect view of the microstructure of nervous tissue in addition to the pathway of the fibres^10^,^11^,^12^,^13^. The diffusion data can help determine the quantitative diffusion values, such as the ADC and fractional anisotropy (FA) values that reflect the directionality of the molecular diffusion. FA values range from zero to one, with high FA values indicating anisotropic diffusion and low FA values indicate increased isotropic diffusion. DTI may be used for the evaluation and visualization of peripheral nerves as well as the measurement of axonal regeneration. A decrease in the mean FA values occurs in injured nerves undergoing demyelination.

DTI has been widely used in the clinic to evaluate lumbar nerve roots. Significant changes in the diffusion parameters of compressed lumbar nerve roots have been reported for patients suffering from disc herniation^7^,^10^,^11^,^14^,^15^. However, few studies reported an association between the DTI parameters and clinical symptoms, and there is only one study that reported DTI changes in compressed nerve roots after removing the herniated lumbar disc^12^. We previously reported that the mean FA value of the compressed lumbar nerve roots was significantly lower than the FA of the contralateral nerve roots and that a significant negative association existed between the FA values and both the Oswestry Disability Index (ODI) and symptom duration in compressed nerve roots^14^,^15^. Eguchi *et al*.^12^ found that among 13 patients (three patients with nerve root compression at L2, L3 or S1; five patients at L4 and five patients at L5), the FA values significantly increased after surgical treatment, and the ADC values decreased but not significantly. Strong correlations between the DTI parameters and the severity of the neurological symptoms were also found. However, the DTI parameters at different locations of the nerve root can be variable. To the best of our knowledge, no previous study has assessed the association between the FA and ADC values and severity and duration of neurological symptoms after surgery in the unilateral nerve root. Consequently, the aim of this study was to evaluate changes in the FA and ADC values after surgery and to investigate the correlation between these changes and the severity and duration of neurological symptoms in patients with unilateral radiculopathic pain caused by LDH.

## Subjects and Methods

### Patients

The study includes 30 patients (eighteen men and twelve women, median age of 40.4 years (21 to 58)) who were treated by micro endoscopic discectomy for unilateral radiculopathy and lumbar disc herniation between May 2015 and May 2016. All patients underwent DTI scanning before and at least 1 month later after surgery. This study was approved by the review board of the Three Gorges University. All study methods were conducted in accordance with the Three Gorges University guidelines and regulations. In addition, all experimental protocols were approved by the Three Gorges University committee. All subjects signed an informed consent form to allow their clinical data to be used for research. The diagnosis of the patients was based on neurological symptoms, plain radiographs, Computed Tomography (CT) images and MRI. The location of the symptomatic nerves in all the patients was at unilateral sacral1 (S1) nerve root. The exclusion criteria were the following: bilateral symptoms and lumbar canal stenosis, previous surgery to the lumbar spine and presents of another spine-related disease. Clinical symptoms were evaluated using an ODI questionnaire for each patient before and at least one month later after surgery, and the duration of the clinical symptoms was based on the earliest incidence of leg pain and numbness. Changes in the FA, ADC and ODI were calculated using the following formula: postoperative FA/ADC/ODI − preoperative score FA/ADC/ODI.

### MRI protocol

A 1.5-T MRI scanner (GE Healthcare, Chalfont St. Giles, United Kingdom) was used in this study. A standard MRI protocol was performed, which included both T1-weighted turbo spin echo (TSE) (repetition time (TR), 660 ms; echo time (TE), 9.5 ms; number of averages (NEX), 1; field of view (FOV), 380 × 380 mm; matrix, 128 × 128; slice count, 12; slice thickness, 5 mm; slice gap, 0.4 mm; acquisition time, 2 min 53 s) and T2-weighted TSE (TR, 2960 ms; TE, 70 ms; NEX, 2; FOV, 380 × 380 mm; matrix, 512 × 512; slice count, 12; slice thickness, 4 mm; slice gap, 0.4 mm; acquisition time, 3 min 21 s) sequences that imaged the lumbar spine in the sagittal plane and a T2-weighted TSE (TR, 5680 ms; TE, 123 ms; FOV, 200 × 200 mm; matrix, 512 × 512; NEX, 2; slice count, 30; slice thickness, 3 mm; slice gap, 0; acquisition time, 3 min 40 s) sequence performed in the axial plane that explored the last two mobile levels of the lumbar spine.

**Figure 1:**
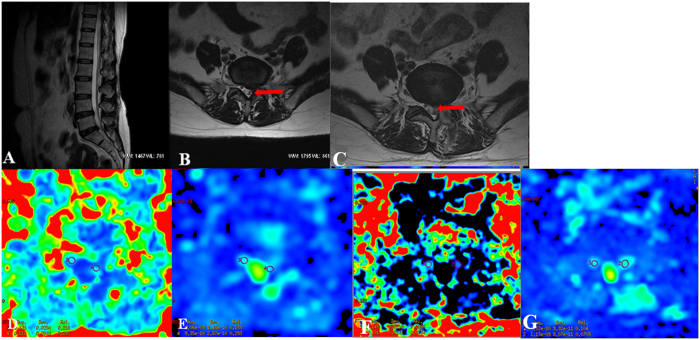
FA and ADC measurements before and after surgery in patients with unilateral S1 disc herniation. The S1 nerve root was compressed on the left side. Bilateral nerve roots were assessed by DTI before (**A**/**B**) and after surgery (**C**). (**D**) shows the FA value before surgery (left side: 0.176, right side: 0.265); (**E**) shows the FA value after surgery (left side: 0.239, right side: 0.268); (**F**) shows the ADC value (10^−3^ mm^2^/s) before surgery (left side: 1.353, right side: 1.113); (**G**) shows the ADC value (10^−3^ mm^2^/s) after surgery (left side: 1.226, right side: 1.122). S, sacral. ROIs are indicated with circles in D/E/F/G.

A single-shot echo-planar spin-echo DTI sequence was performed in the axial plane from the L4-S2 interbody spaces with the following parameters (Fig. 1): TR, 8400 ms; TE, 89.1 ms; FOV, 240 × 240 mm; matrix, 128 × 128; NEX, 4; slice count, 23; slice thickness, 3 mm; slice gap, 0; b value, 900 s/mm^2^; motion probing gradients applied in 15 directions; acquisition time, 9 min 01 s. The range of the DTI scanning in this study was from the lower half of lumbar vertebral 4 to sacral 2, which covered the entire S1 nerve root.

### Image analysis

**Figure 2:**
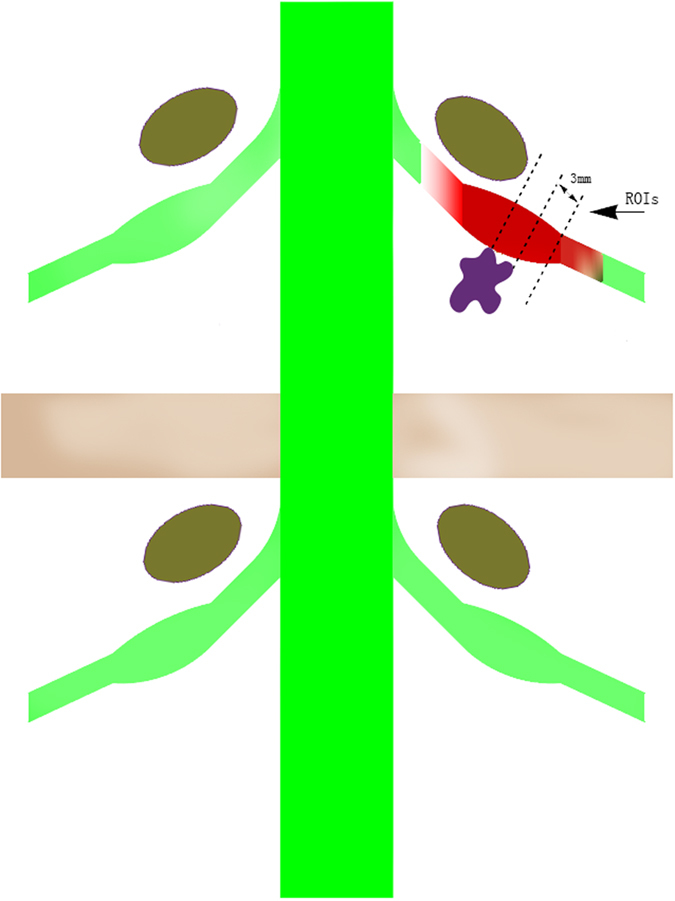
Diagram showing that the regions of interest (ROIs) were designated at three levels at 3-mm intervals proximal to the site of nerve compression due to disc herniation on the axial image.

All MRI scans were reviewed and agreed upon by two radiologists blinded to the clinical data. All DTI analyses were performed by one radiologist and one trained spinal surgeon. The image analysis was independently performed for each participant immediately after acquisition for qualitative assessment and data extraction. The diffusion data sets were corrected for motion-induced artefacts using the Automated Image Registration package. This software and the image registration algorithms within it are widely used in the DTI community to register and estimate DTI indices for spinal cord imaging. Motion correction of the diffusion-weighted images was performed using the rigid scaled-least-squares method. The effects of cerebrospinal fluid contamination were low because the slice thickness was 3 mm smaller than the size of the S1 dorsal root ganglion, which were 5 mm in width and 10 mm in length. To avoid the muscle fibre bundle, water in hollow organs and other signal interference, the lower limit of the FA threshold value was set to 0.1, and the upper limit was set to 0.5. Since the b value ranges from 0 to 1000 s/mm^2^, neurography could avoid images with obvious artefacts with a b value (900 s/mm^2^) when a DTI scan was performed, which is based on previous studies. After motion correction of the diffusion-weighted images, gradient directions for these images were rotated based on the registration. Eigenvectors and eigenvalues of the diffusion tensor matrix from the axial images were then computed on a voxel-by-voxel basis. Anatomical axial T2 and DTI images were merged. Anatomical fusion between the axial T2 sequence and the DTI reconstructions was performed to allow better visualization of the different anatomic spaces. Measurements were focused on the site of the compression before and after surgery (Fig. 1). The relative location of the region of interest (ROI) before surgery was marked relative to the vertebral body and pedicle on both the compressed and contralateral sides of each patient. The postoperative ROI was identified relative to the preoperative marked sites (Fig. 2). The ROI was circular with a diameter of 3 voxels; the average volume of the ROI was equal at the compressed nerve roots before and after surgery. The ROIs were set at 3 levels of the compressed nerve root as shown in Fig. 2. The size of ROIs, ranging from 20 to 45 mm^2^, was selected to accurately include the respective nerve roots while limiting the presence of other tissues to avoid partial volume effects when calculating the mean FA and ADC. The diffusion tensor fields were diagonalized to obtain eigenvalues and eigenvectors for each voxel. The eigenvector associated with the largest eigenvalue was used to represent the main direction.

### Statistical analysis

Statistical analysis was conducted using a standard SPSS 16.0 (SPSS Institute, Chicago, IL) software package. All values are presented as the mean and standard deviation (SD). A paired samples t-test was performed to compare the compressed side before and after surgery. Pearson correlation coefficients were calculated to determine the correlation between the DTI parameters and intensity and duration of the symptoms. A threshold of p < 0.05 was considered statistically significant.

### Ethics approval and consent to participate

The approval of the Three Gorges University review boards was obtained before conducting the present study. All subjects signed informed consent form, allowing their clinical data to be used for research.

## Results

**Figure 3:**
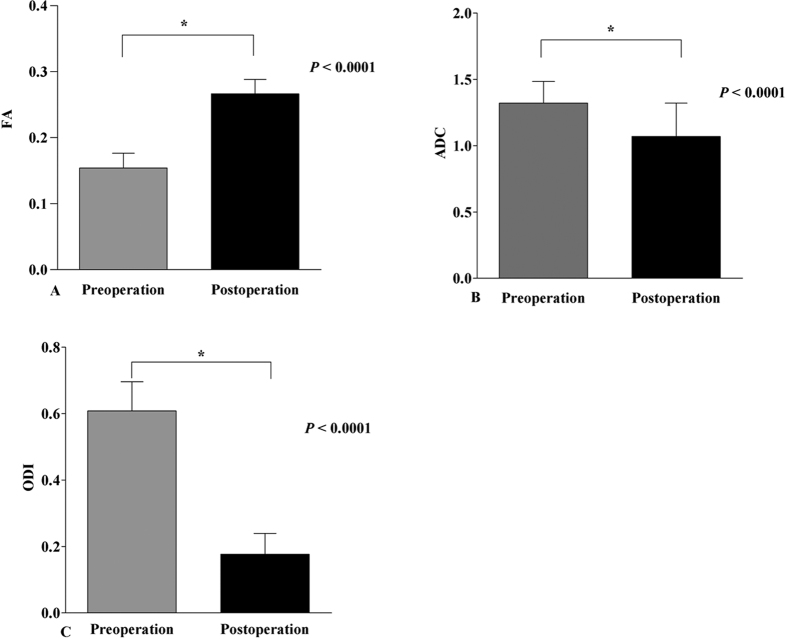
FA/ADC/ODI values of the compressed nerve roots before and after surgery in patients with unilateral S1 disc herniation.

The mean duration of the clinical symptoms was 1.93 months. The preoperative and postoperative FA values were 0.154 and 0.267, respectively. The FA values were increased significantly at 1 month after surgical treatment (p < 0.0001) (Fig. 3A). The preoperative and postoperative ADC values (10^−3^ mm^2^/s) were 1.32 and 1.07, respectively. The preoperative and postoperative ODI values (expressed as a decimal) were 0.608 and 0.177, respectively. Both the ADC and ODI values were noticeably decreased at 1 month after surgical treatment (p < 0.0001) (Fig. 3B and C).

**Figure 4:**
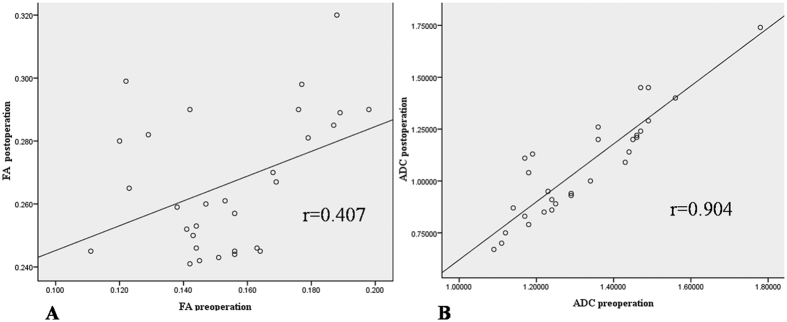
There was a significant positive correlation between the preoperative FA/ADC values and postoperative FA/ADC values (P < 0.05 and P < 0.000, respectively).

**Figure 5:**
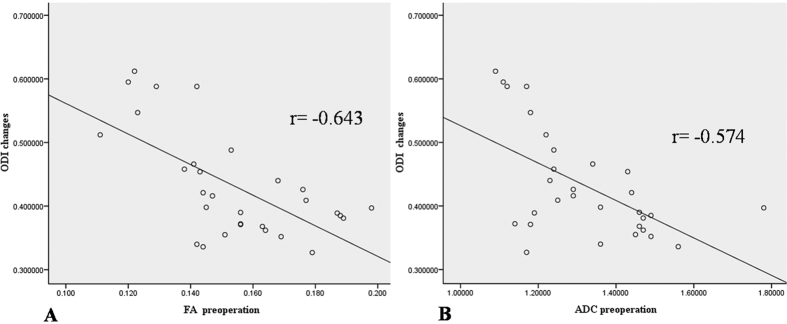
There was a strong negative correlation between the preoperative FA/ADC values and ODI changes (P < 0.000 and P < 0.000, respectively).

**Figure 6:**
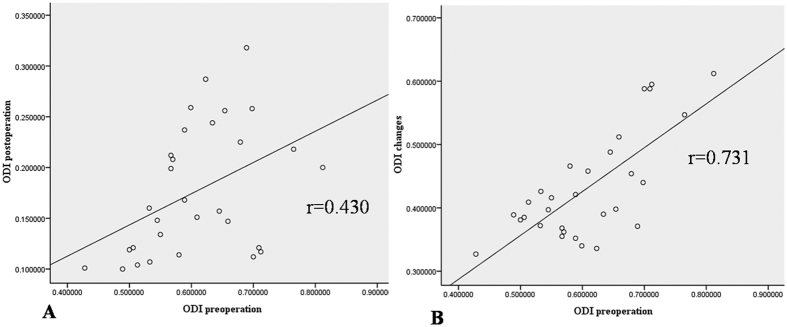
There was a significant positive correlation between the preoperative ODI values and both ODI changes and the postoperative ODI values (P < 0.000 and P < 0.05, respectively).

**Figure 7:**
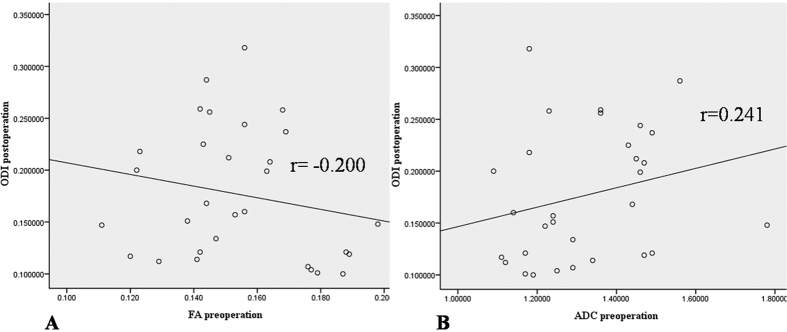
There was no correlation between the postoperative ODI values and the preoperative DTI parameters (P > 0.05).

**Figure 8:**
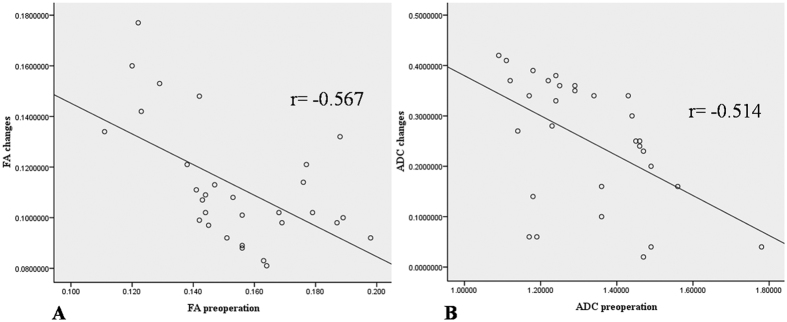
There was a strong negative correlation between the preoperative DTI parameters and changes in the DTI parameters after surgery (P < 0.000 and P < 0.000, respectively).

**Figure 9:**
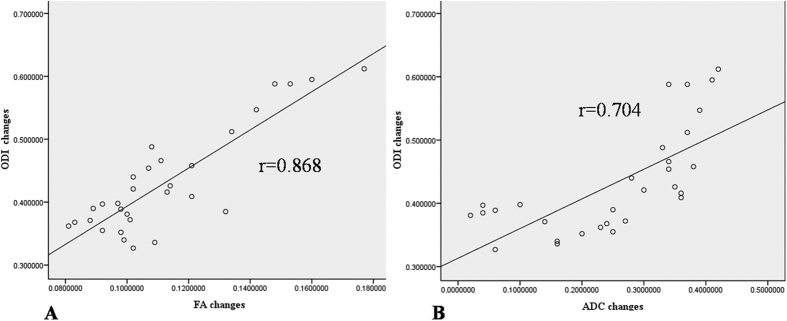
There was a significant positive correlation between changes in the DTI parameters and ODI changes after surgery (P < 0.000 and P < 0.000, respectively).

**Figure 10:**
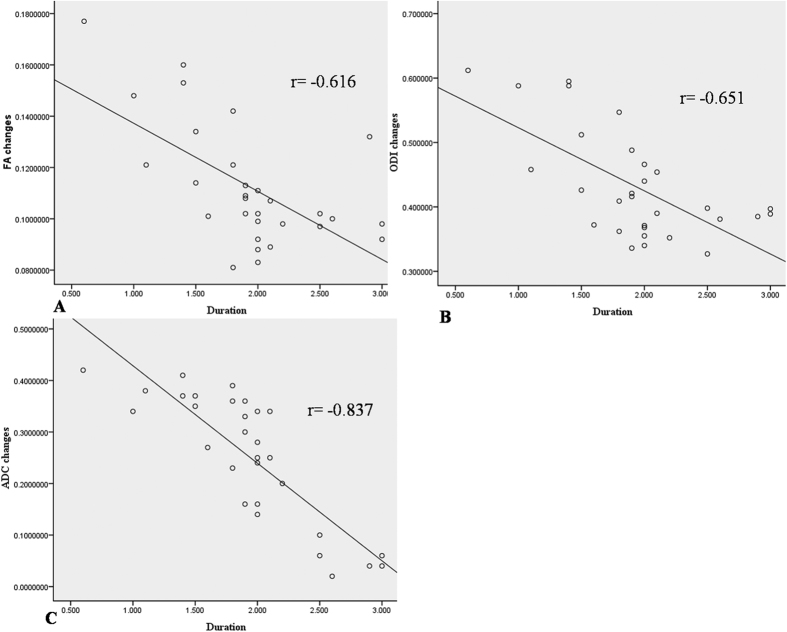
There was a strong negative correlation between symptom duration and changes in both the DTI parameters and ODI after surgery (P < 0.000, P < 0.000, and P < 0.000, respectively).

All the correlations are summarized in Table 1. The analysis showed no significant correlation between ODI and duration (r = 0.063, p = 0.741). There was a strong positive correlation between the preoperative and postoperative DTI parameters (FA: r = 0.407, p = 0.025; ADC: r = 0.904, p < 0.0001) (Fig. 4A and B). There was a strong negative correlation between the preoperative DTI parameters and changes in the ODI (FA: r = −0.643, p < 0.0001; ADC: r = −0.574, p = 0.001) (Fig. 5A and B). There was a strong positive correlation between the preoperative ODI and both the postoperative ODI (r = 0.430, p = 0.018) and ODI changes (r = 0.731, p < 0.0001) (Fig. 6A and B). There was no significant correlation between the preoperative DTI parameters and the postoperative ODI (FA: r = −0.200, p = 0.292; ADC: r = 0.241, p = 0.199) (Fig. 7A and B). There was a strong negative correlation between the preoperative DTI parameters and changes in the DTI parameters (FA: r = −0.567, p = 0.001; ADC: r = −0.514, p = 0.004) (Fig. 8A and B). There was a strong positive correlation between the changes in the DTI parameters and ODI changes (FA: r = 0.868, p < 0.0001; ADC: r = 0.704, p < 0.0001) (Fig. 9A and B). There was a strong negative correlation between symptom duration and both changes in the DTI parameters (FA: r = −0.616, p < 0.0001; ADC: r = −0.837, p < 0.0001) (Fig. 10A and C) and ODI changes (r = −0.651, p < 0.0001) (Fig. 10B). The lumbar nerves from a 41-year-old man with disc herniation at L5/S1 before and after surgery is shown in Fig. 1.

## Discussion

Mechanical compression and chemical insult can induce various histological changes in the lumbar nerve roots, including the acceleration of vascular permeability with a disrupted nerve root barrier and the development of subperineurial or intraneural edema in the nerves. These microstructural changes alter water diffusion along the nerve by increasing the distance between the axons and axon fascicles. If the compression is not relieved quickly, the nerve root will sustain hypoxia- ischaemia injury; this causes symptoms such as back pain and leg numbness, which can influence daily activities. Although LDH nerve root compression in patients is well visualized using MRI, its correlation with clinical symptoms is still controversial. As the DTI technique has evolved, the movement of water molecules can be accurately measured using DTI parameters such as the FA and ADC values. These values have been found to be capable of analysing the nerve root condition in greater detail than conventional MRI^10^,^11^,^12^,^14^.

For patients with LDH who do not achieve good recovery with conservative treatment, surgical interventions, which include micro endoscopic discectomy, discectomy with insertion of pedicle screws, and percutaneous transforaminal endoscopic discectomy, should be considered^16^,^17^,^18^. However, the microstructural improvement of compressed nerve roots is critical to ensure surgical success. Although the DTI technique is widely used in peripheral nerves such as nerve roots in the lumbar region, those assessments focused on normal or preoperative individuals, and there were only two studies regarding the changes in the microstructure of compressed nerve roots after surgery in patients with LDH, including our previous study^12^,^15^. In clinical practice, re-examining the quality of DTI after surgery is often diminished by the metal instrument fixating on the vertebral pedicles and surrounding tissue edema. Therefore, the present study implemented a surgical procedure using percutaneous transforaminal endoscopic discectomy, and the second DTI scan was performed at least one month after surgery. The present study also reported both the FA value and ADC value, which showed significant differences at the compressed side before and after surgery, and those values were consistent with those of normal nerve roots as shown in our previous findings. There may be many aspects to the mechanism of DTI recovery. After surgical removal of the herniated disc as well as reduction of subperineurial and intraneural edema, blood flow and ischaemia of the compressed nerve roots were gradually improved. Microstructural changes before surgery such as demyelination of the nerve fibres, Wallerian degeneration, and endoneurial fibrosis could be partially or mostly recovered. Therefore, water diffusion along the nerve is either normal or near normal.

Although a close relationship between clinical symptoms and the DTI parameters in patients with LDH has been strongly suggested, whether DTI can be used to predict functional recovery after surgery has not been determined^12^,^14^,^15^. Eguchi reported strong correlations between the DTI parameters and the severity of the neurological symptoms before surgery, and a strong correlation between the ADC value and leg numbness after operation was also found. In that study, only 13 patients were included, and the compressed nerve roots were located at different segments^12^. Gao *et al*.^19^ reported that the FA values were positively correlated with clinical symptoms in patients with myelopathy due to cervical spondylosis, which was consistent with our findings. Jones *et al*. reported that the preoperative FA at the level of stenosis correlated with improvement of clinical symptoms after surgery^13^. Wen *et al*. also reported that FA correlated with improvement of the modified Japanese Orthopaedic Association score^20^. In the present study, a strong positive correlation between the preoperative DTI parameters and the postoperative DTI was found, which indicates that microstructural recovery of a compressed nerve root after surgery depends on preoperative parameters. Our result also showed a strong positive correlation between the preoperative ODI and both postoperative ODI and ODI changes, which suggests that the preoperative clinical symptoms have an important influence on the postoperative values. Moreover, the present study found a strong positive correlation between changes in the DTI parameters and ODI changes, indicating that the microstructural changes of the compressed nerve root were consistent with symptoms in LDH patients after surgery. Therefore, DTI may be a promising modality to predict functional recovery after surgery. The present results also showed a strong negative correlation between the preoperative DTI parameters and ODI changes. This means that if the ADC values were high, the patients were more likely to have reduced improvement after surgery, and if the FA values were low, the patients had a better outcome. According to our previous results, the preoperative FA values were positively associated with the preoperative ODI, and the FA/ADC values of the compressed nerve roots after surgery were not significantly different from those of normal nerve roots, which indicated that the lower the preoperative FA, the more severe the patients’ condition. Therefore, a negative correlation between the preoperative FA values and ODI changes was not observed. However, some studies (including our previous study) indicated that ADC was not a an accurate parameter to assess nerve injury. We will investigate whether there exists a correlation between the preoperative ADC values and ODI changes in future studies.

There is no consensus regarding the timing of elective surgery for the treatment of LDH. Previous observational studies have identified worse outcomes in patients with a longer duration of symptoms prior to treatment and have recommended earlier surgical intervention^21^,^22^,^23^. Jansson *et al*. showed that in LDH patients who underwent surgery, symptom duration longer than six months was a risk factor for a worse health-related quality of life^24^. Ng *et al*. found that patients with symptoms for more than twelve months had obviously worse outcomes than patients with symptoms for less than twelve months prior to operative treatment^25^. In addition, Peul *et al*.^26^ indicated that early surgery offered significant benefits during the follow-up period over nonoperative treatment. Pitsika showed significant improvement in patients with symptoms beyond 1 as well as 2 years since disease onset after viable surgery^23^. The present results showed that the longer duration, the lower FA and ADC improvement, which indicated that symptom duration was an important factor to predict the microstructural recovery of the nerve root after surgery. Additionally, our study found a strong negative correlation between symptom duration and ODI changes, which showed that the duration could effectively predict clinical recovery.

There are several limitations to this study. First, the number of patients was small, and further prospective analyses with a larger patient cohort are required to confirm its reproducibility. Second, the two-month follow-up was short. Third, there was no control group. However, the postsurgical DTI values in this study were as same as those in our previously study in which DTI was used to assess normal nerve roots in patients with LDH. Fourth, the same ROIs that were manually designated before and after surgery may affect the DTI values.

In conclusion, we found that the FA values were significantly decreased in the symptomatic nerves but subsequently increased two months after surgery, which correlated well with the ODI scores and symptom duration. The ADC values were significantly decreased in symptomatic nerves after surgery and were also correlated with the ODI scores and symptom duration. DTI parameters such as FA can predict the improvement of clinical symptoms after surgery, thus making these values a potential tool for the functional diagnosis of lumbar nerve damage.

## Additional Information

**How to cite this article**: Wu, W. *et al*. Microstructural changes are coincident with the improvement of clinical symptoms in surgically treated compressed nerve roots. *Sci. Rep.* **7**, 44678; doi: 10.1038/srep44678 (2017).

**Publisher's note:** Springer Nature remains neutral with regard to jurisdictional claims in published maps and institutional affiliations.

## Figures and Tables

**Table 1:** The correlations between DTI parameters and ODI and duration.

r value	ODI Post-	ODI changes	ADC Post-	ADC changes	FA Post-	FA changes
FA-Pre	−0.200	−0.643			0.407	−0.567
FA-Post		0.297				
FA-changes		0.868			0.522	
ADC-Pre	0.241	−0.574	0.904	−0.514		
ADC-Post		−0.723				
ADC-changes		0.704	−0.832			
ODI-Pre	0.430	0.731	−0.527		−0.154	
ODI−Post			−0.219		−0.698	
Duration		−0.651	0.788	−0.837	0.061	−0.616

FA: fractional anisotropy; ADC: apparent diffusion coefficient; Pre: pre-operation; Post: post-operation; ODI: Oswestry Disability Index.

## References

[b1] Majeed S. A. Seshadrinath N. A. Binoy K. R. & Raji L. Lumbar disc herniation: Is there an association between histological and magnetic resonance imaging findings? Indian journal of orthopaedics 50, 234–242 (2016). 10.4103/0019-5413.181794 PMC4885290 27293282

[b2] Brayda-Bruno M. . Advances in the diagnosis of degenerated lumbar discs and their possible clinical application. European spine journal: official publication of the European Spine Society, the European Spinal Deformity Society, and the European Section of the Cervical Spine Research Society 23, 315–323 (2014). 10.1007/s00586-013-2960-9 23978994

[b3] Wang Y. J. Towards consistency for magnetic resonance (MR) relaxometry of lumbar intervertebral discs. Quantitative imaging in medicine and surgery 6, 474–477 (2016). 27709087 10.21037/qims.2016.08.04 PMC5009100

[b4] Mostofi K. & Karimi Khouzani R. Reliability of the Path of the Sciatic Nerve, Congruence between Patients’ History and Medical Imaging Evidence of Disc Herniation and Its Role in Surgical Decision Making. Asian spine journal 9, 200–204 (2015). 25901230 10.4184/asj.2015.9.2.200 PMC4404533

[b5] Aragao Homem C. Fonseca A. C. Geraldes R. & Pinho e Melo T. Brain Magnetic Resonance with Negative Diffusion-Weighted Imaging: Does It Preclude Acute Stroke Diagnosis? Journal of stroke and cerebrovascular diseases: The official journal of National Stroke Association 24, 251–253 (2015). 10.1016/j.jstrokecerebrovasdis.2015.04.038 26169550

[b6] Sakai T. . Diffusion-weighted imaging and diffusion tensor imaging of asymptomatic lumbar disc herniation. The journal of medical investigation: JMI 61, 197–203 (2014). 24705766 10.2152/jmi.61.197

[b7] Takashima H. . Efficacy of diffusion-weighted magnetic resonance imaging in diagnosing spinal root disorders in lumbar disc herniation. Spine 38, 998–1002 (2013). 10.1097/BRS.0b013e31829862d3 23632334

[b8] Basser P. J. & Jones D. K. Diffusion-tensor MRI: theory, experimental design and data analysis - a technical review. NMR in biomedicine 15, 456–467 (2002). 12489095 10.1002/nbm.783

[b9] Backens M. Basic principles and technique of diffusion-weighted imaging and diffusion tensor imaging. Der Radiologe 55, 762–770 (2015). 26330214 10.1007/s00117-015-0004-7

[b10] Manoliu A. . Diffusion Tensor Imaging of Lumbar Nerve Roots: Comparison Between Fast Readout-Segmented and Selective-Excitation Acquisitions. Investigative radiology 51, 499–504 (2016). 26895194 10.1097/RLI.0000000000000260

[b11] Li J. Wang Y. Wang Y. Lv Y. & Ma L. Study on lumbosacral nerve root compression using DTI. Biomedical reports 5, 353–356 (2016). 27602215 10.3892/br.2016.734 PMC4998170

[b12] Eguchi Y. . Diffusion tensor imaging of radiculopathy in patients with lumbar disc herniation: preliminary results. The bone & joint journal 98, 387–394 (2016). 26920965 10.1302/0301-620X.98B3.36036

[b13] Jones J. G. Cen S. Y. Lebel R. M. Hsieh P. C. & Law M. Diffusion tensor imaging correlates with the clinical assessment of disease severity in cervical spondylotic myelopathy and predicts outcome following surgery. American journal of neuroradiology 34, 471–478 (2013). 22821918 10.3174/ajnr.A3199 PMC7965104

[b14] Wu W. . Microstructural Changes in Compressed Nerve Roots Are Consistent With Clinical Symptoms and Symptom Duration in Patients With Lumbar Disc Herniation. Spine 41, 661–666 (2016). 26656057 10.1097/BRS.0000000000001354

[b15] Wu W. . Microstructural changes in compressed nerve roots treated by percutaneous transforaminal endoscopic discectomy in patients with lumbar disc herniation. Medicine 95, 5106; 10.1097/MD.0000000000005106 (2016). PMC5059094 27749591

[b16] Gadjradj P. S. van Tulder M. W. Dirven C. M. Peul W. C. & Sanjay Harhangi B. Clinical outcomes after percutaneous transforaminal endoscopic discectomy for lumbar disc herniation: a prospective case series. Neurosurgical focus 40, 10.3171/2015.10.FOCUS15484 (2016). 26828884

[b17] Yeo C. G. . Three-Years Outcome of Microdiscectomy via Paramedian Approach for Lumbar Foraminal or Extraforaminal Disc Herniations in Elderly Patients over 65 Years Old. Korean Journal of Spine 13, 107–113 (2016). 27799988 10.14245/kjs.2016.13.3.107 PMC5086460

[b18] Zhao C. Q. Ding W. Zhang K. & Zhao J. Transforaminal lumbar interbody fusion using one diagonal fusion cage with unilateral pedicle screw fixation for treatment of massive lumbar disc herniation. Indian journal of orthopaedics 50, 473–478 (2016). 27746488 10.4103/0019-5413.189595 PMC5017167

[b19] Gao S. J. . Correlation study of 3T-MR-DTI measurements and clinical symptoms of cervical spondylotic myelopathy. European journal of radiology 82, 1940–1945 (2013). 23932097 10.1016/j.ejrad.2013.06.011

[b20] Wen C. Y. . Is diffusion anisotropy a biomarker for disease severity and surgical prognosis of cervical spondylotic myelopathy? Radiology 270, 197–204 (2014). 10.1148/radiol.13121885 23942607

[b21] Rihn J. A. . Duration of symptoms resulting from lumbar disc herniation: effect on treatment outcomes: analysis of the Spine Patient Outcomes Research Trial (SPORT). The Journal of bone and joint surgery American 93, 1906–1914 (2011). 10.2106/JBJS.J.00878 PMC5515548 22012528

[b22] Rothoerl R. D. Woertgen C. & Brawanski A. When should conservative treatment for lumbar disc herniation be ceased and surgery considered? Neurosurgical review 25, 162–165 (2002). 10.1007/s101430100184 12135229

[b23] Pitsika M. Thomas E. Shaheen S. & Sharma H. Does the duration of symptoms influence outcome in patients with sciatica undergoing micro-discectomy and decompressions? The spine journal: official journal of the North American Spine Society 16, 21–25 (2016). 10.1016/j.spinee.2015.12.097 26940192

[b24] Jansson K. A. Németh G. Granath F. Jönsson B. & Blomqvist P. Health-related quality of life in patients before and after surgery for a herniated lumbar disc. The Journal of bone and joint surgery British 87, 959–964 (2005). 10.1302/0301-620X.87B7.16240 15972911

[b25] Ng L. C. & Sell P. Predictive value of the duration of sciatica for lumbar discectomy. A prospective cohort study. The Journal of bone and joint surgery British 86, 546–549 (2004). 15174551

[b26] Peul W. C. . Surgery versus prolonged conservative treatment for sciatica. The New England journal of medicine 356, 2245–2256 (2007). 17538084 10.1056/NEJMoa064039

